# Cases of Cremation

**Published:** 1874-12

**Authors:** 


*November:*

Cases of Cremation.—The body of the late lady Dilke, wife of the well-known liberal, Sir Charles Dilke, was taken to Dresden and burned last month, at her own urgent request before death. The ceremony was performed in the furnace recently invented for burial purposes by Herr Siemens, and the relatives of the deceased lady permitting strangers to be present, a large number of scientific men attended the experiment. When the company had complied with Herr Siemen's request to offer up a mental prayer, the coffin was placed in the chamber of the furnace; six minutes later the coffin burst; five minutes more and the flesh began to melt away; ten minutes more and the skeleton was laid bare; another ten minutes and the bones began to crumble. Seventy-five minutes after the introduction of the coffin into the furnace, all that remained of lady Dilke and the coffin were six pounds of dust, placed in an urn.

This was October 10. On September 22, in Breslau, another corpse was burned, the furnaces of the city gas works being used. According to the *Medicinische Central Zeitung*, it required two hours and ten minutes to reduce it to ashes. These weighed three pounds. It is gratifying to learn that only two hectolitres of coke were required, the total cost of which was thirteen and two-tenths silver groschen, or thirty-one cents United States currency! This is literally cheaper than dirt, and the grave-digger's occupation will soon be gone.

